# Xinyang flavivirus, from Haemaphysalis flava ticks in Henan Province, China, defines a basal, likely tick-only Orthoflavivirus clade

**DOI:** 10.1099/jgv.0.001991

**Published:** 2024-05-29

**Authors:** Lan-Lan Wang, Qia Cheng, Natalee D. Newton, Michael T. Wolfinger, Mahali S. Morgan, Andrii Slonchak, Alexander A. Khromykh, Tian-Yin Cheng, Rhys H. Parry

**Affiliations:** 1 College of Veterinary Medicine, Hunan Agricultural University, Changsha, Hunan Province, PR China; 2 Children’s Medical Center, Hunan Provincial People’s Hospital, Changsha, PR China; 3 School of Chemistry and Molecular Biosciences, The University of Queensland, Brisbane, QLD, Australia; 4 Australian Infectious Diseases Research Centre, The University of Queensland, Brisbane, QLD, Australia; 5 Department of Theoretical Chemistry, University of Vienna, Vienna, Austria; 6 Research Group Bioinformatics and Computational Biology, Faculty of Computer Science, University of Vienna, Vienna, Austria; 7 Bioinformatics Group, Department of Computer Science, University of Freiburg, Freiburg, Germany; 8 RNA Forecast e.U., Vienna, Austria; 9 GVN Center of Excellence, Australian Infectious Diseases Research Centre, Brisbane, QLD, Australia

**Keywords:** Binjari virus, chimeric virus, *Flavivirus*, *Haemaphysalis flava*, orthoflavivirus, structural RNA, tick-borne orthoflavivirus, tick-borne pathogen, xrRNA

## Abstract

Tick-borne orthoflaviviruses (TBFs) are classified into three conventional groups based on genetics and ecology: mammalian, seabird and probable-TBF group. Recently, a fourth basal group has been identified in *Rhipicephalus* ticks from Africa: Mpulungu flavivirus (MPFV) in Zambia and Ngoye virus (NGOV) in Senegal. Despite attempts, isolating these viruses in vertebrate and invertebrate cell lines or intracerebral injection of newborn mice with virus-containing homogenates has remained unsuccessful. In this study, we report the discovery of Xinyang flavivirus (XiFV) in *Haemaphysalis flava* ticks from Xìnyáng, Henan Province, China. Phylogenetic analysis shows that XiFV was most closely related to MPFV and NGOV, marking the first identification of this tick orthoflavivirus group in Asia. We developed a reverse transcriptase quantitative PCR assay to screen wild-collected ticks and egg clutches, with absolute infection rates of 20.75 % in adult females and 15.19 % in egg clutches, suggesting that XiFV could be potentially spread through transovarial transmission. To examine potential host range, dinucleotide composition analyses revealed that XiFV, MPFV and NGOV share a closer composition to classical insect-specific orthoflaviviruses than to vertebrate-infecting TBFs, suggesting that XiFV could be a tick-only orthoflavivirus. Additionally, both XiFV and MPFV lack a furin cleavage site in the prM protein, unlike other TBFs, suggesting these viruses might exist towards a biased immature particle state. To examine this, chimeric Binjari virus with XIFV-prME (bXiFV) was generated, purified and analysed by SDS-PAGE and negative-stain transmission electron microscopy, suggesting prototypical orthoflavivirus size (~50 nm) and bias towards uncleaved prM. *In silico* structural analyses of the 3′-untranslated regions show that XiFV forms up to five pseudo-knot-containing stem-loops and a prototypical orthoflavivirus dumbbell element, suggesting the potential for multiple exoribonuclease-resistant RNA structures.

## Data Availability

Raw high throughput sequencing read files are archived at the NCBI Sequencing Read Archive database under BioProject ID PRJNA280697 and accession number SRR1958881. The Xinyang flavivirus genome has been deposited in Genbank under the accession OP699738.

## Introduction

The genus *Orthoflavivirus* (family *Flaviviridae*) exhibits a notable ecological diversity, traditionally classified into four distinctive categories: vector-borne, vertebrate-infecting mosquito (MBF) or tick (TBF) borne orthoflaviviruses, which can cause disease in a wide range of vertebrates, including humans, exemplified by the type species yellow fever virus (YFV) [1,2]. Separate from vector-borne orthoflaviviruses (VBFs), there are two lineages of insect-specific orthoflaviviruses which exclusively replicate in insect cells. The first lineage are classic orthoflaviviruses (cISFs), and the second lineage is dual-host associated orthoflaviruses (dISFs) which are closely related the VBFs but also cannot replicate in mammalian cells [3,4]. Lastly, there is a group of orthoflaviviruses found in mammals with no known arthropod vector, referred to as the NKV group [5].

*Orthoflavivirus* members have positive-sense RNA genomes encoding a polyprotein that is post-translationally cleaved by viral and host proteases into three structural proteins [capsid (C), pre-membrane (prM) and envelope (E)] and seven non-structural (NS) proteins (NS1, 2A, 2B, 3, 4A, 4B, 5) [6,7]. Additionally, *Orthoflavivirus* genomes feature a 5′ untranslated region (UTR) of ~100 nt and a 3′ UTR of ~400–700 nt, facilitating viral RNA replication and translation. These regions also contain highly structured elements such as exoribonuclease-resistant RNAs [8].

The International Committee on Taxonomy of Viruses has classified 12 species of TBFs into three groups: the mammalian host-TBF group, which includes tick-borne encephalitis virus (TBEV), Gadgets Gully virus, Kyasanur Forest disease virus, Langat virus, louping ill virus, Omsk haemorrhagic fever virus, Powassan virus and Royal Farm virus; the seabird host-TBF group, comprising Meaban virus, Saumarez Reef virus and Tyuleniy virus; and the probable-TBF group, represented solely by Kadam virus [1,9] (ICTV Chapter on *Orthoflavivirus* available at: https://ictv.global/report/chapter/flaviviridae/flaviviridae/orthoflavivirus). Tick-borne orthoflaviviruses persist through a multifaceted enzootic transmission cycle requiring the blood meal of a tick from a vertebrate host [2].

Mpulungu flavivirus (MPFV) was recently identified in *Rhipicephalus muhsamae* ticks from Zambia [10], and is distantly genetically related to these three groups. The MPFV genome shares genetic similarity to fragments of Ngoye virus (NGOV), identified in *R. evertsi* and *R. guilhoni* ticks from Senegal [11]. Despite extensive efforts, MPFV and NGOV have not been isolated from tick homogenates through propagation in the vertebrate cell lines baby hamster kidney (BHK-21), African green monkey kidney (VeroE6), amphibian *Xenopus* (XTC) or human (SW13). Attempts in acarid *Ixodes scapularis* (ISE6), *R. appendiculatus* (RAE25) and mosquito *Aedes albopictus* (C6/36) cell lines also yielded no successful isolations. Additionally, no virus was recoverable in intracerebral injection into neonatal mice with MPFV and NGOV. This lack of replication in vertebrate or invertebrate cells is unusual, given that tick orthoflaviviruses have historically been propagated and cultured using these methods [12,13]. The failure to isolate MPFV and NGOV through these techniques might suggest a unique ecological niche within the genus *Orthoflavivirus*, perhaps as arthropod-specific viruses with a limited capacity to infect vertebrates.

Genus- and family-wide evolutionary analyses of *Orthoflavivirus* members have indicated that MPFV is basal to the vector-borne tick, mosquito orthoflaviviruses and NKV orthoflaviviruses [14,15], supporting the proposed emergence of vector-borne orthoflaviviruses from a Chelicerata or acarid host [15].

In China, TBEV is endemic to the northeast, northwest and southwest regions [16]. In the northeast Heilongjiang Province, laboratory-confirmed TBEV is a notifiable disease and has been identified in the neighbouring Jilin and Liaoning Provinces as well as Inner Mongolia [17]. Phylogenetic analyses based on Chinese TBEV isolates and E amplicons indicated that all belong to the Far-Eastern TBEV subtype [18].

In light of extensive tick virus diversity and incidence, including TBEV, uncovered through metagenomic sequencing efforts in China [19,24], we sought to further understand the diversity of tick-borne orthoflaviviruses in the country. To achieve this, we conducted high-throughput metatranscriptomics and subsequent screening of wild samples.

Here we assemble and describe a near-complete genome of a novel orthoflavivirus from *Haemaphysalis flava* ticks (Acari: Ixodidae) sampled from Xìnyáng, Henan Province, China which we have named Xinyang flavivirus (XiFV), suggested species name *Orthoflavivirus xinyangense. H. flava* is a ubiquitous tick species widely distributed in East Asia with a host range that encompasses small mammals such as dogs, goats, hedgehogs and the giant panda [25,26]. Moreover, *H. flava* can also parasitize humans and various migratory and indigenous birds [27]. While *H. flava* ticks have previously been identified as potential vectors of TBEV in the Republic of Korea [28,30], there are no reports of TBEV-positive *H. flava* ticks in China [17]. To enhance our understanding of the prevalence and transmission of XiFV, we developed a reverse transcriptase quantitative PCR (RT-qPCR) screening assay of wild-caught samples. Additionally we generated a chimeric Binjari virus containing the prM-E of XiFV (bXiFV) to examine general structural features of the virus under SDS-PAGE and negative-stain transmission electron microscopy (TEM).

## Methods

### Field site selection and sample processing

Ticks were collected from wild European hedgehogs (*Erinaceus europaeus*), from Xìnyáng (32° 13′ N 114° 08′ E), Henan Province, China. To ensure accurate identification, morphological features [31] were observed under stereomicroscopy and molecular analysis methods such PCR of the COX1 and ITS1 genes of mitochondrial DNA (mtDNA). PCR of samples and comparisons against *H. flava* mitochondrion (GenBank: MG604958) were employed [32]. For initial metatranscriptome assembly, 50 salivary glands were dissected as previously described [33]. These samples were stored in liquid nitrogen until RNA extraction. For wild screening of samples, engorged ticks were sexed and female engorged ticks were maintained in individual plates until they laid egg clutches, as previously described [34]. Subsequently, both the adult tick and the egg clutch were washed with sterile PBS and flash frozen until downstream processing.

### RNA extraction and high-throughput sequencing

Extraction of total RNA from tick salivary glands was carried out using TRIzol Reagent (Invitrogen). Total RNA concentrations were calculated using a NanoDrop spectrophotometer (Thermo Scientific) and the Agilent Bioanalyzer 2100 (Agilent Technologies). A total of 10 µg RNA extracted from salivary gland samples was subjected to further purification using the RNeasy kit (Qiagen). Subsequent mRNA purification, and first- and second-strand synthesis for library sequencing preparation were as described previously [33]. For library sequencing preparation, a 100 bp paired-end sequencing approach was employed on the HiSeq 2500 platform conducted by Bohao Biotechnology Corporation (China).

### RNA extraction, RT-PCR screening and 5’/3′ RACE of XiFV-positive samples

For screening of wild-caught samples to detect XiFV, RNA extraction was perfomed using the TaKaRa MiniBEST Viral RNA/DNA Extraction Kit (Takara Bio). First-strand cDNA was synthesized utilizing TransScript One-Step cDNA Synthesis SuperMix (TransGen Biotech). PCR amplification of a 516 bp XiFV NS3 fragment from cDNA with the PCR primers XiFV_F (5′-CAAGCTAGGAAGAACTATGAGGTGG-3′) and XiFV_R (5′-CTCACTCATCACCACCATGTCCT-3′) was performed under the following cycling conditions: 5 min (95 °C); 35 cycles of 30 s (95 °C), 30 s (59.5 °C) and 60 s (72 °C); 10 min (72 °C). For samples confirmed as XiFV positive, 5′ RACE was undertaken with the primer XiFV_5′ (5′- CGTCAAGGTAGCTGAATGTACTCCAC-3′) under the following cycling conditions: 5 min (95°C); 35 cycles of 30 s (95°C), 30 s (61°C) and 60 s (72°C); 10 min (72°C). PCR products were separated in 1 % agarose gel and purified using the Wizard SV Gel and PCR Clean-Up System (Promega). Purified PCR products were cloned into pGEM-T, transformed into competent DH5α cells and sent to Beijing Genome Institute for Sanger sequencing.

### RT-qPCR assay of wild-caught samples for viral copy number quantification

For the qPCR assays, the PerfectStart Green qPCR SuperMix (+Dye I/+Dye II) (TransGen Biotech) was employed. These assays were conducted using the StepOne Real-Time PCR System (Thermo Fisher Scientific). The RT-qPCR primers employed were XiFV-qF (5′-CATCACTTTCACGAGGTTCGCTTG-3′) and XiFV-qR (5′-CTGCCTATCAGTTCATCCTGGTCC-3’), for an amplicon size of 79 bp. Reaction conditions were an initial pre-denaturation step at 95 °C for 30 s, 40 cycles of denaturation at 95 °C for 5 s and specific primer annealing at 60 °C for 34 s. A standard curve for XiFV genomic DNA (gRNA) was constructed through serial dilution of the standard sample prepared in triplicate.

### *De novo* assembly and phylogenetic position of Xinyang flavivirus

Basecalled fastq files had low-quality and adapter sequences removed using Cutadapt v1.21 [35] and assembled using rnaviralSPAdes v3.15.4 [36]. Assembled contigs were queried against a virus protein database [37] using BLASTx. For coverage statistics, fastq basecalled files were mapped to the assembled XiFV contig using Bowtie2 v2.2.7 [38]. Per-base coverage of the XiFV genome ws determined using samtools v1.3, as previously described [39].

For phylogenetic placement of XiFV within the genus *Orthoflavivirus*, 75 polyprotein sequences, including partial NGOV and Jiutai virus, were aligned using MAFFT v7.490 with the L-INS-i method and BLOSUM45 matrix [40], resulting in a multiple sequence alignment (MSA) of 76×3993 positions. Gblocks v0.91b was used remove ambiguously aligned regions from the MSA [41]. A consensus maximum-likelihood phylogenetic tree was reconstructed using IQ-TREE2 v2.1.2, employing the LG+F+R7 protein substitution model selected with ModelFinder [42]. The consensus tree was generated with ultrafast bootstrap and SH-aLRT test (--alrt 1000 -B 1000) and visualized using Figtree v1.4.4 (A. Rambaut; https://github.com/rambaut/figtree/releases).

### Xinyang flavivirus genomic functional annotation

The XiFV polyprotein was predicted with the NCBI ORF finder tool (https://www.ncbi.nlm.nih.gov/orffinder/). To characterize the domains of the XiFV polyprotein we queried the sequence with InterProScan (v5.60-92.0) [43]. For predicted cleavage residues, we used the polyprotein MSA and previously determined cleavage sites of TBEV/MPFV to rationally align NS3-Pro motifs alongside the transmembrane topology prediction using DeepTMHMM (v1.0.24) [44]. For putative signal peptides, a window of 50 aa up- and downstream of the aligned MPFV polyprotein was examined using SignalP v6 [45]. Furin cleavage analysis was undertaken using the ProP-1.0b Server (https://services.healthtech.dtu.dk/services/ProP-1.0/). To analyse dinucleotide motifs of the coding region of the XiFV genome, we used a dataset of dinucleotide motif odds ratios from 94 *Orthoflavivirus* genomes [46,47] and partial NGOV sequences (GenBankID: DQ400858.1, EU074038.1). Hierarchical clustering and principal components analysis (PCA) were performed using ClustVis [48], with values being log(*x*+1) transformed for analyses.

### Stuctural modelling of the E and prM of XiFV

For structural analyses, we employed ColabFold v1.5.2 (AlphaFold2) to model envelope glycoproteins E and prM of XiFV templates from the pdb70 database and MMseqs2 under default conditions. Due to the databases having bias towards mature forms of E, we predicted an immature E as a prM:E heterodimer [49] using the alphafold2_multimer_v3 modelling settings. For each prediction five top models, ranked based on a per-residue confidence score, were manually inspected using ChimeraX [50]. Model 1 was chosen for E (mature monomer) and prM (immature). Model 4 of immature E was selected from the prM:E heterodimer prediction with prM removed due to the positioning of the transmembrane regions. The trimer was arranged using PDB:7L30.

### Generation of BinJ/XiFV-prME (bXiFV) recombinant virus

Chimeric infectious DNA constructs between BinJV (GenBankID: MG587038.1) and XiFV were generated by circular polymerase extension reaction (CPER) as previously described [51,53]. Briefly, the prME of XiFV with BinJV overlaps was ordered as gene fragments (Twist Bioscience). The XiFV gene fragment was PCR amplified using primer pair XiFV-prME-F (5′-GCTGCTCGTTGGAGCAGGAGCGATGGCT-3′) and XiFV-prME-R (5′-CATTTCTTTTCGGCTAATGTCCAGACTGCATCCTATTTC-3′). PCR products were separated in 1 % agarose gel, and extracted using a Monarch DNA Gel Extraction Kit (NEB).

The CPER assembly utilized GXL polymerase (Takara Bio) to generate circularized DNA from 0.1 pmol of each viral fragment, as well as a fragment containing the optimized OpIE2-CA insect promotor [54]. Thermocycling conditions were 13 cycles with denaturation at 98 °C, annealing at 60 °C and extension at 68 °C for 12 min, with a final additional extension at 68 °C for 12 min. The CPER was transfected into *Aedes albopictus* C6/36 cell monolayers (ATCC CRL1660) maintained in RPMI 1640 supplemented with 2 % FBS, 1 % GlutaMAX (Gibco) and 100 U ml^−1^ penicillin–streptomycin (Gibco) at 28 °C, using TransIT-LT1 (Mirus) as per the manufacturer’s instructions. Passage 0 (P_0_) transfected cells and supernatant were harvested at 7 days post-transfection, and reseeded into a T75 flask (P_1_). Supernatant and cells from P_1_ was blind passsaged (P_2_) onto C6/36 cells. P_1_ and P_2_ stocks were titrated via an immunoplaque assay using anti-NS1 monoclonal 4G4 (Mozzy MABs, University of Queensland, Australia) in C6/36 cells with titres of 10^3.79^ and 10^5.23^ f.f.u. ml^–1^, respectively. To generate sufficient bXiFV for electron microscopy, sub-confluent monolayers of C6/36 cells in ten T175 flasks were infected by P_2_ bXiFV stock at an m.o.i. of 0.001. Supernatant was harvested at 4, 6, 8 days and, after each collection, cells were replenished with fresh RPMI containing 2 % FBS. Final pooled virus culture supernatant (P_3_) was clarified by centrifugation at 3000 r.p.m. for 30 min at 4 °C and vaccum filtered through a 0.22 µm filter. bXiFV virions were precipitated via addition of polyethylene glycol (PEG 8000, 8 %) and stirred slowly overnight at 4 °C. PEG-precipitated virus was pelleted at 12 000 r.p.m. (Beckman Coulter JLA 10.500 rotor) for 1.5 h at 4 °C, before ultracentrifugation through a sucrose cushion at 28 000 r.p.m. for 2 h (Beckman Coulter SW32 Ti rotor). The pellet was incubated overnight at 4 °C before density gradient purification using a potassium tartrate gradient (25–40 %) at 50 000 r.p.m. for 1 h at 4 °C (Beckman Coulter SW60 Ti) as previously described [55,56]. Purified virus was collected, and buffer exchanged into NTE buffer using a 30 kDa Amicon filter and stored at 4 °C. Purified virus was quantified using NanoDrop One (Thermo Fisher Scientific) and 10 µg was run on SDS-PAGE with Bolt 4–12 % Bis-Tris plus gels (Invitrogen); total protein was then observed through Coomassie stain, destained and imaged on a Bio-Rad GelDoc Go imaging system (Bio-Rad Laboratories). To ensure fidelity of the recombinant bXiFV, RNA was extracted from the P_2_ and P_3_ viral stocks using the Nucleospin RNA virus kit (Macherey-Nagel). This RNA was then used with the previously mentioned XiFV-prME-F/R primer pair in conjunction with the SuperScript III One-Step RT-PCR kit (Invitrogen). After RT-PCR, samples were examined using gel electrophoresis, gel purified as above and sent for Sanger sequencing at the Australian Genome Research Facility.

### Negative stain electron microscopy

Purified bXiFV was diluted to 2 mg ml^−1^ in NTE buffer, before adsorbing 4 µl onto carbon glow-discharged formvar-coated 200-mesh copper grids (ProSciTech) for 2 min. The grids were blotted and washed three times with water and stained with 2 % uranyl acetate solution, with blotting in between. The grids were imaged using a Hitachi HT7700 transmission electron microscope operated at 100 kV.

### Prediction of RNA secondary structure

For RNA secondary structure predictions within the XiFV UTRs, we employed the RNAfold and RNALfold tools from the Vienna RNA Package v2.6.4 [57] and pKiss v2.2.13 [58]. We used Infernal [59] covariance models (CMs) to assess the structural homology of the predicted evolutionarily conserved elements with similar elements in other orthoflaviviruses. Additionally, we verified the predicted loci of exoribonuclease-resistant RNAs (xrRNAs), a dumbbell (DB) element and the terminal stem-loop (3′SL) element in the XiFV 3′ UTR using TBF-specific CMs from two recent studies [60,61]. As the XiFV 3′ UTR was incompletely sequenced, we used the terminal 52 nt from MPFV to model the XiFV 3′SL element. All secondary structure plots were generated using VARNA v3.93 [62], employing varnaapi (https://pypi.org/project/varnaapi/).

## Results

### Discovery of a novel orthoflavivirus in *Haemaphysalis flava* ticks from Henan Province, China

**Fig. 1.**
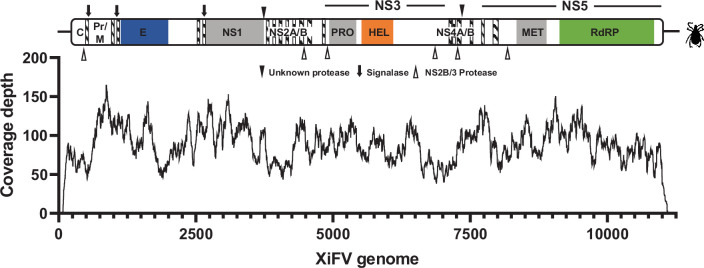
Genome architecture and coverage depth of Xinyang flavivirus (XiFV). Structural orthoflavivirus domains: capsid protein C (PF01003), propeptide pr (PF01570), envelope glycoprotein M (PF01004) and E (PF00869 and PF21659). Non-structural proteins: NS1 (PF00948), NS2A/B (PF01002). The NS3 serine protease domain PRO (PF00949) and helicase HEL (PF07652, PF20907). The NS4A/B (PF01350 and PF01349) and the NS5 methyltransferase MET (PF01728) and the NS5 RdRP domain (PF00972 and PF20483). Diagonal hatched lines indicate predicted transmembrane domains. Polyprotein cleavage sites are marked according to the key.

Salivary glands collected from semi-engorged, wild-caught *H. flava* samples were subject to high-throughput transcriptome sequencing [33]. Following quality control, a total of 36 million paired-end reads were *de novo* assembled. Virus-like contigs were then identified using a previously described pipeline [37]. One 11 056 nt contig with an average coverage of ~87.9× and constituting 0.01 % of the total library was identified through BLASTx analysis as a probable *Orthoflavivirus* (Fig. 1). This contig exhibited a high nucleotide identity, 90.93 %, with an unpublished 916 nt NS5 fragment of Jiutai virus identified from *Haemaphysalis japonica* ticks from Jilin, China, in 2018 (GenBankID: MT246200.1). The closest match with a published complete genome was Mpulungu flavivirus [10] (GenBankID: LC582740.1), sharing 68.85 % identity over 37 % of the query coverage (BLASTn, E-value: 1E-50). This level of similarity was comparable to a 4176 nt polyprotein fragment of NGOV [11] (GenBankID: DQ400858.1, Identity: 71.98 %, Query cover: 12 %, E-value: 9E-44). Given the geographical origin of the sample, we tentatively named this virus XiFV and suggest the species name *Orthoflavivirus xinyangense*. To obtain more of the genome, RACE revealed an additional 49 nt in the 5′ UTR, while attempts to determine more of the 3′ UTR were unsuccessful.

### The polyprotein of XiFV contains prototypical orthoflavivirus features but lacks a furin cleavage site in prM

Our final recovered XiFV genome has a single ORF encoding a polyprotein that is 3408 aa in length with 5′ and 3′ UTRs of 140 and 660 nt, respectively. BLASTp analysis of the predicted polyprotein suggests the closest full-length polyprotein hits were Mpulungu flavivirus with 65.58 % identity (GenBankID: BCL56285.1, Query cover: 100 %, E-value: 0) and Kyasanur Forest disease virus with 44.30 % identity (GenBankID: AXB87763.1, Query cover: 100 %, E-value: 0).

We predicted the potential NS2B-NS3-protease and host signalase sites for processing the polyprotein of XiFV and closely related *Orthoflavivirus* members. Biochemically the NS3-protease of almost all orthoflaviviruses cleaves preferentially after two basic amino acid residues (RR/RK/KR) followed by a small amino acid (G/A/S). For XiFV, all predicted NS3-protease cleavage sites conform to the canonical NS3-protease motifs (Table S1, available in the online version of this article). For orthoflaviviruses the pre-membrane (prM) protein is cleaved by the host convertase furin into propeptide (pr) and membrane (M) [63], at the conserved motif R-X-R/K-R. However, both XiFV and MPFV lack a canonical furin cleavage site at the aligned prM cleavage regions (Fig. S1). Specifically, for XiFV, the region is 192-RIVERSLSVT-201 with a ProP score of 0.157, while for MPFV, it is 186-RIAERSLSVT-195 with a ProP score of 0.138. In contrast, TBEV possesses an optimal furin cleavage site (201-SRTRR↓SVLIP-210) with a ProP score of 0.788. No alternative furin cleavage sites could be predicted within a 40–50 aa sliding window for both XiFV and MPFV (Fig. S1).

**Fig. 2.**
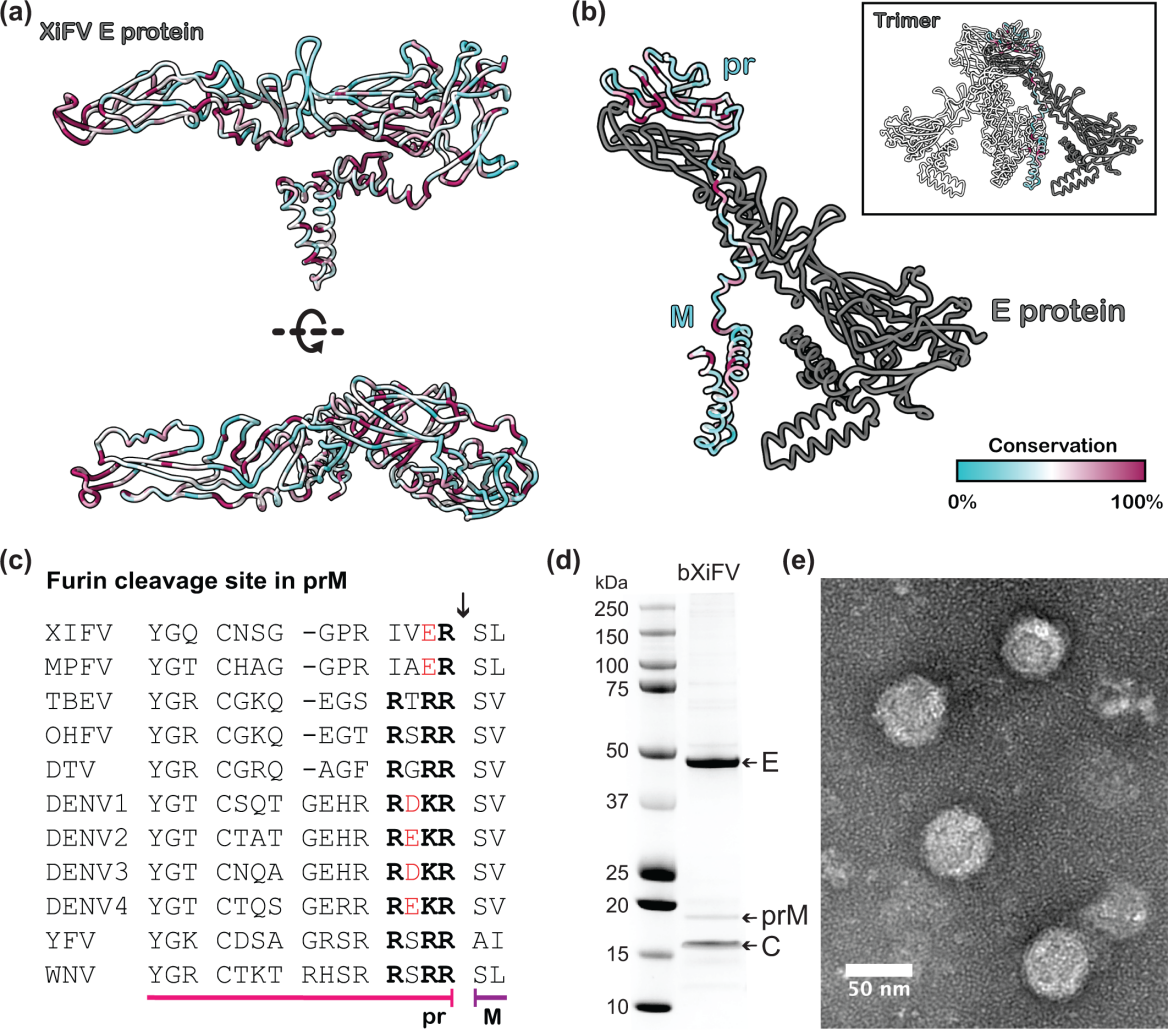
Features of XiFV structural proteins. (a) Cartoon representation of the predicted Alphafold2 XiFV E monomer in the mature form. Residues are coloured according to the conservation of amino acids across the E protein between members of the family *Flaviviridae* (Fig. S2). (**b**) Predicted Alphafold2 XiFV prM and E heterodimer in the immature conformation. Residues of prM are coloured according to the conservation of amino acids across the prM protein between members of the family *Flaviviridae* (Fig. S2). E is coloured in grey. Insert shows predicted prM and E in the trimer form. (**c**) Alignment of XiFV and MPFV prM at the furin cleavage site. Arrow indicates the cleavage site. Acidic residues are indicated by red and basic residues indicated at the furin cleavage site (R-X-R/K-R) are in bold. Viruses used for this alignment include TBEV (GenBankID: ABS00284), DTV (GenBankID: AF311056), OHFV (GenBankID: QRI43507), DENV1 (GenBankID: AYP31257), DENV2 (GenBankID: QIJ58805), DENV3 (GenBankID: QIS48879), DENV4 (GenBankID: AVY51410), YFV (GenBankID: QGN18670) and WNV (GenBankID: QKN22593). (**d**) SDS-PAGE (4–12 %) analysis of gradient purified bXIFV virions stained with Coomassie protein stain. (**e**) Negative-staining transmission electron micrograph of bXIFV particles following potassium tartrate gradient purification and staining with 2 % uranyl acetate.

The alignment of XiFV prM and E with other tick-borne orthoflaviviruses shows conserved orthoflavivirus features with amino acid similarity of 25–33 % across prM and 35–47 % across E. This conservation strongly suggests that the virion structure of XiFV would be similar to other tick orthoflaviviruses. Modelling of the E glycoprotein of XiFV using ColabFold yielded a structure of E in its mature dimeric form (Fig. 2a). This model exhibited the characteristic features of a orthoflavivirus E protein with three domains: DI, the central beta-barrel domain; DII, the elongated dimerization domain; and DIII, the immunoglobulin-like domain, followed by a stem and transmembrane domain at the C-terminus. The fusion peptide, an essential component for viral membrane fusion [64,65], was predicted to be located at the distal end of DII and displayed a high degree of conservation across XiFV and MPFV (Fig. S2). In general, there are more conserved residues (coloured maroon in Fig. 2) on the internal side of E compared to surface exposed regions, which is typical of other orthoflaviviruses [66].

Due to the absence of an optimal furin cleavage site in prM, the trimeric form of prM and E of XiFV was also predicted using ColabFold multimer modelling and independently fitted to a trimeric prM:E model (PDB:7L30). As anticipated, the predicted structure of trimeric XiFV prM and E has pr capping the tip of the E protein spike (Fig. 2b). The interface between pr and E contained a region characterized by conserved residues (Asp57–Cys65), further indicating that XiFV is likely to share structural similarities with other immature orthoflaviviruses [55,67].

To further validate whether XiFV structural proteins (prM and E) would form orthoflavivirus-like virions, the prM and E genes of XiFV were used to generate a chimera with the backbone (5′ and 3′ UTRs, capsid, NS1-5) of the insect-specific orthoflavivirus, Binjari virus [52]. This chimera (bXiFV) was successfully recovered from C6/36 mosquito cells and gradient purified. Concentrated virions were assessed by SDS-PAGE (Fig. 2d), which revealed distinct bands at approximately 48, 18 and 16 kDa which correlate with the protein sizes of E, prM and C, respectively. There are less prominent bands between 10 and 15 kDa, which may indicate low levels of cleavage from unknown proteases or the presence of cellular proteins as additional bands are also present at >50 kDa. Additionally, the purified bXiFV virions were imaged by negative-stain TEM, which shows XiFV prM and E form typical orthoflavivirus-like enveloped virions approximately 50 nm in diameter (Fig. 2e).

### XiFV forms a well-supported basal clade to the mosquito-borne and tick-borne orthoflaviviruses

**Fig. 3.**
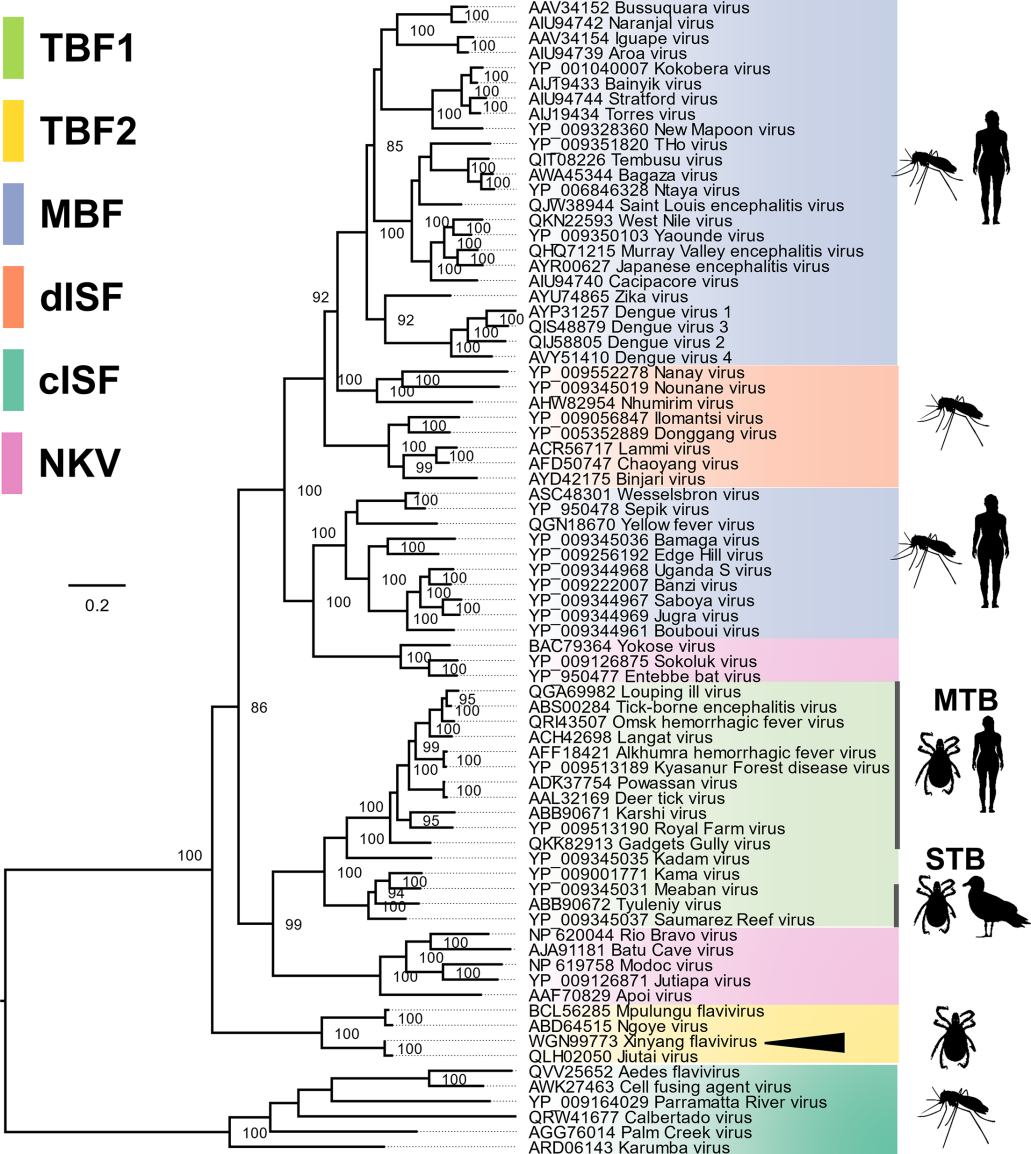
Xinyang flavivirus groups phylogenetically to the basal tick-only orthoflavivirus group or tick-borne orthoflavivirus group two (TBF2). Maximum clade credibility phylogeny of XiFV, indicated with an arrowhead, within the genus *Orthoflavivirus*. The tree is midpoint rooted and groups are coloured as per the key. Branch length represents amino acid substitutions per site. The mammalian host-TBF (MTB) and the seabird host-TBF (STB) groups are shown within the tick-borne orthoflavivirus group one (TBF1). Nodes with bootstrap support <85 are not shown.

To elucidate the phylogenetic position of XiFV within the genus *Orthoflavivirus*, 75 representative complete polyprotein sequences and fragments of NGOV and Jiutai virus were aligned using MAFFT. We constructed a maximum-likelihood phylogenetic tree based on this alignment using IQ-TREE2 (Fig. 3). The consensus phylogenetic trees placed XiFV in a robustly supported basal clade with MPFV, NGOV and Jiutai virus with the closest clades, by branch length or amino acid divergence, being the seabird host-TBF group. The overall topology of this tree is congruent with previously created phylogenies [15,46, 47, 68]. Recognizing the distinct divergence of this group from other tick-borne orthoflaviviruses, we propose the designation of this clade as tick-borne orthoflavivirus group (TBF2).

### XiFV exhibits dinucleotide composition similar to classical insect-specific orthoflaviviruses

**Fig. 4.**
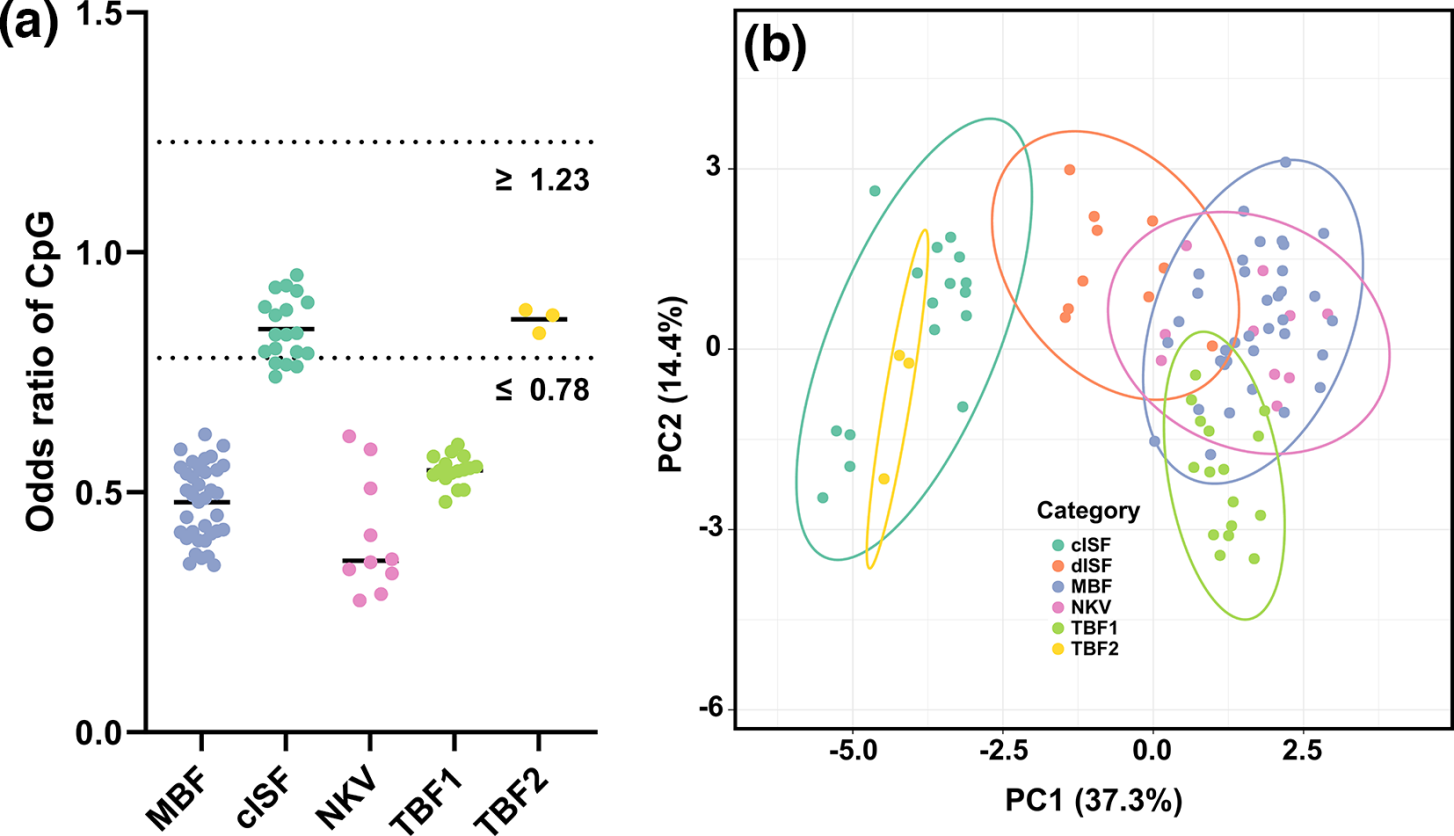
Xinyang flavivirus coding sequence exhibits dinucleotide composition that closely resembles cISFs. (**a**) Odds ratio of CpG in the coding sequence of different orthoflavivirus groups with known host range. (**b**) PCA of the odds ratios of dinucleotides of the coding sequence. Original values have been log(*x*+1)-transformed. Rows are scaled to unit variance, and principal components are calculated using Singular Value Decomposition with imputation. Prediction ellipses bound a 95 % probability within the assigned group. Data points are coloured as follows: cISF (classical insect-specific orthoflavivirus), dISF (dual-host associated, or lineage insect-specific orthoflavivirus), MBF (mosquito-borne orthoflavivirus), NKV (no known vector orthoflavivirus), TBF1 (tick-borne orthoflavivirus group one) and TBF2 (tick-borne orthoflavivirus group two).

The vertebrate antiviral protein ZAP is known to bind viral RNA containing CpG, effectively inhibiting viral replication [69]. Notably, all vertebrate-infecting orthoflaviviruses demonstrate underrepresentation of CpG dinucleotides in their genomes [70], whereas cISFs do not exhibit selection against CpG dinucleotides. Odds ratios are used to quantify the expected dinucleotide composition relative to observed ratios. When there is no significant selection against a motif, the odds ratio approaches 1. Statistically significant underrepresentation or overrepresentation is indicated by odds ratios ≤0.78 or ≥1.23, respectively. For XiFV, the odds ratio of the CpG motif is 0.83, which is consistent with the odds ratios of CpG observed for MPFV and NGOV, the other tick-borne orthoflavivirus group two (TBF2) members. TBF2 members share a CpG composition similar to cISFs (*n*=19). In contrast vertebrate-infecting TBF1 (*n*=16), no known vector orthoflaviviruses (NKVs, *n*=12) and mosquito-borne orthoflavivirus group members (MBF, *n*=36) all exhibit statistically significant CpG underrepresentation (Fig. 4a).

Recognizing that single dinucleotide motifs can be overly simplistic, we employed a comprehensive analysis by assessing the frequencies of each dinucleotide, resulting in 16 parameters for clustering analyses. Specifically, we performed PCA (Fig. 4b) and hierarchical clustering (Fig. S3) to assess the potential natural groupings between the two tick-borne orthoflavivirus groups (TBF1/TBF2) and other ecological groups within the genus *Orthoflavivirus*. The results of PCA (Fig. 4b) indicate TBF2 clusters completely within the probability prediction ellipses of the cISFs. This pattern was consistent when examining the dendrogram derived from hierarchical clustering analysis (Fig. S3), with all TBF2 viruses clustering within the cISF groupings. Collectively, these findings underscore the similarity in dinucleotide composition between TBF2 coding sequences and cISFs, setting them apart from the vertebrate-infecting TBF1 group. This, in conjunction with the phylogenetic divergence of XiFV, further solidifies the separation between these two distinct tick-borne orthoflavivirus groups.

### RT-PCR analysis of wild-collected ticks and egg clutches reveals potential adult female to egg transmission of XiFV

To assess the natural incidence of XiFV in wild populations of *H. flava,* we developed an RT-qPCR screen based on the assembled XiFV genome. This screening was carried out on engorged ticks collected from European hedgehogs (*Erinaceus europaeus*), from Xinyang, Henan Province, China, between 2015 and 2022. Our sampling included adult male and females, as well as larvae and nymphs. Engorged adult female ticks were allowed to generate egg clutches within the laboratory setting. Subsequently, random samples were selected, and each tick was triturated for RT-qPCR-based infection determination. We utilized a standard curve, with C_t_ values >30 indicating less than one genome copy per reaction (5 μl), which served as the threshold for infection. Melt curve examination was undertaken for positive samples.

The results, summarized in Table 1, revealed that only female adult ticks and egg samples tested positive for XiFV, with infection rates of 20.75 and 15.31 %, respectively. Specifically, the presence of XiFV viral RNA in egg samples suggests that infected female ticks can pass the virus to their offspring during embryogenesis, highlighting a potentially crucial aspect of the potential transovarial virus lifecycle within tick populations. While these findings indicate that XiFV can be passed from adult female ticks to egg clutches, further studies are required to assess the extent of transovarial transmission of XiFV in *H. flava* ticks.

**Table 1.** qRT-PCR positivity rate of individual *Haemaphysalis flava* ticks and egg clutch samples

Samples	No. of samples	RT-PCR-positive samples	Infection rate (%)
Female tick	138	22	20.75
Male tick	96	0	0
Nymphs	124	0	0
Larval tick	88	0	0
Eggs	98	15	15.31

### Analysis of the XiFV 3′ UTR reveals up to five potentially pseudo-knot-forming stem loops and a prototypical orthoflavivirus dumbbell element

**Fig. 5.**
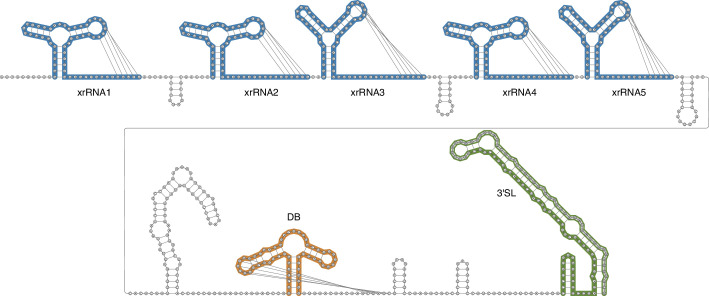
Predicted secondary structure of the XiFV 3′ UTR showing the overall architecture of structured RNAs. Evolutionarily conserved elements are highlighted in colour: five pseudoknot-forming three-way junction xrRNAs (blue), a dumbbell (DB) element (orange), and a 3′ stem-loop (3′SL) element (green). For modelling the 3′SL, the terminal 52 nt of the MPFV genome were used (depicted in grey). No evolutionary support was observed for the stem-loop element located between the xrRNAs and the DB element.

During orthoflavivirus infection, the host 5′−3′ exoribonuclease Xrn1 degrades viral gRNA, reviewed by Slonchak and Khromykh [8]. To counter the enzymatic activity of Xrn1, all known orthoflaviviruses contain evolutionarily conserved xrRNAs within their 3′ UTRs [71]. This results in the production of smaller RNA genome fragments call sub-genomic flaviviral RNAs (sfRNAs) and, as a consequence, these sfRNAs accumulate within infected cells [8]. Due to the dynamic folding and refolding of these structural RNA element duplications, diversity and redundancies of xrRNAs are common, especially for insect-specific orthoflaviviruses, allowing the production of different species of sfRNAs [68]. *Orthoflavivirus* xrRNAs are short RNA molecules with lengths ranging from 60 to 90 nt. They commonly exhibit a three-way junction structure, where the apical hairpin loop forms a pseudoknot, referred to as PK2. PK2 interacts with a sequence region downstream of the three-way junction structure. For TBF1 xrRNAs, RNA structure probing experiments have suggested a base-pair span of between 30 and 40 nt for PK2 [72]. Our model of the XiFV 3′ UTR predicts five putative xrRNA elements (Fig. 5). Covariance model analysis suggests that these elements are structurally homologous to previously described TBF1 xrRNAs [61]. However, it is worth noting a distinct feature: the closing stem of the XiFV xrRNA three-way junction is shorter, and more closely resembles MFV xrRNAs [72]. This shortened closing stem directly influences the extension of PK2, which is predicted to span between 20 and 30 nt. This observed divergence in xrRNA structures between TBF2 and TBF1, along with the unique similarity of XiFV xrRNA’s stem to MBF, can probably be attributed to the genetic divergence and phylogenetic relationship between these groups. Given that XiFV is basal to the vector-borne orthoflavivirus clades, it is plausible that the xrRNA structures found in TBF2 might be ancestral forms of this structure. Following the xrRNAs, the XiFV 3′ UTR contains a prototypical DB element, which exhibits structural homology to the *Orthoflavivirus* DB element (Rfam Accession: RF00525, Bit score: 42.2, E-value: 4.5e-8). Interestingly, the proximal hairpin of the XiFV DB elements can form a pseudoknot with a sequence region immediately downstream of the element, a feature also described for MBFs [73]. The XiFV 3′ UTR is predicted to terminate with a canonical 3′SL element, as known from other orthoflaviviruses. To achieve a prototypical 3′SL structure, we utilized the terminal 52 nt from MPFV due to incomplete sequencing of the XiFV genome.

## Discussion

In this study, we report a novel orthoflavivirus, provisionally named XiFV, from *H. flava* ticks in China. Our approach involved RNA sequencing and RACE techniques, which allowed us to determine the complete coding sequence of the polyprotein, along with nearly complete 5′ and 3′ UTRs. XiFV is most closely related to an unpublished fragment of another orthoflavivirus, Jiutai virus, an orthoflavivirus found in *Haemaphysalis japonica* ticks from Jilin, China, in 2018, despite a geographical separation of approximately 1635 km. The presence of two closely related tick orthoflaviviruses in separate *Haemaphysalis* species raises the possibility that this tick orthoflavivirus group may have a broader distribution across China and Asia.

Beyond China, XiFV’s closest genetic relatives have been identified in *Rhipicephalus* ticks including MPFV, discovered in Zambia [10], and NGOV, detected in Senegal [11]. The discovery of XiFV, MPFV and NGOV hints at the possibility of a worldwide distribution for this proposed tick-borne orthoflavivirus group two. Historically, TBFs have been prevalent in North America, Europe and as far east as Siberia, Republic of China, Republic of Korea and Japan [2,16, 74]. The identification of this novel tick-orthoflavivirus clade, potentially restricted to tick hosts, opens the possibility of examining potential virus inhibition effects in vector tick species, similar to co-infections seen in vector-borne orthoflavivirus species with insect-restricted orthoflaviviruses [53,75], and between distantly related arthropod-only viruses and pathogenic orthoflaviviruses [76,77].

Efforts to culture NGOV and MPFV via intracerebral inoculation of newborn mice with tick homogenates and utilizing both vertebrate and invertebrate cells have been unsuccessful, hinting at a unique host range for these tick orthoflaviviruses. Analysis of dinucleotide composition reveals that type two TBFs share dinucleotide and CpG composition similarities with classical insect-only orthoflaviviruses. This characteristic makes them potential targets for the mammalian ZAP protein, suggesting that replication in ZAP competent cells would be unlikely. Previous discriminant analysis of dinucleotide composition has been shown to accurately predict host range in the *Flaviviridae*, with positive prediction rate of the host assignment at 76 % compared to 27 % for a random or background model [78]. This further supports the suggestion that these type two TBFs may be restricted to arthropods, making them a tick-only clade.

Many tick-borne and mosquito-borne orthoflaviviruses have demonstrated vertical transmission, from adult ticks to their offspring, although the transmission frequency is usually too low to solely sustain viruses within the vector populations [79,80]. We show a raw positivity rate of 15.31 % in egg clutches for XiFV, suggesting that vertical transmission might be sufficient to maintain this potential tick-only group within the *Haemaphysalis* tick populations. Additionally, as XiFV-positive adult females were not cross-referenced with their respective egg clutches, the maternal transmission rates might be even higher. Further studies are needed to explore maternal transmission rates.

Furthermore, the presence of potential multiple xrRNAs and complex 3′ UTRs are common features in insect-specific orthoflaviviruses, which have evolved duplications and redundancies to evade Xrn1 exoribonuclease activity on the viral gRNA [68]. A notable characteristic of group two tick-borne orthoflaviviruses is the absence of the furin cleavage motif in the prM protein of XiFV and MPFV, suggesting a possibility for these viruses to exist predominantly in an immature state. This was further validated by recovery and purification of a chimeric XiFV, which revealed the presence of uncleaved prM indicating predominantly immature virions. The immature virion is traditionally seen as the non-infectious form of the virus in orthoflavivirus infection and replication [81]. Interestingly, two dual-host-associated or lineage two insect-specific orthoflaviviruses [67,82] have been reported to exist predominantly in the immature form. Binjari virus primarily adopts the immature orthoflavivirus structure, whereas Dongang virus exhibits a preference for the mature form of the virion, albeit with pr still associated. Most importantly, the immature Binjari virus particle was shown to be an infectious form of the virion, which is contrast to most vertebrate-infecting orthoflaviviruses, indicating some orthoflaviviruses may have alternative maturation and/or infection pathways. Although the furin cleavage site within prM does not solely govern the level of maturity of orthoflavivirus virions, it significantly contributes to this process.

## Conclusion

Understanding the ecology and interactions between vector and vertebrate hosts and viruses is crucial, especially in the context of tick-borne diseases. This knowledge is essential for public health and for developing effective strategies to combat these infections. Metagenomic surveillance and analyses for both unculturable viruses and vector tick species should be incorporated into interdisciplinary [83] efforts to uncover the incidence and diversity of tick-borne disease. While it is unlikely that XiFV is a orthoflavivirus that can infect vertebrates, additional research is required to examine the distribution, host range and potential interactions with other tick orthoflaviviruses. This will help us better understand how XiFV may impact vector competence and transmission cycles of tick-borne disease.

## supplementary material

10.1099/jgv.0.001991 Uncited Supplementary Material 1.

## Abbreviations

C: capsid
cISF: classic orthoflavivirus
CPER: circular polymerase extension reaction
DB: dumbbell
E: envelope
gDNA: genomic DNA
MBF: mosquito-borne orthoflavivirus
MPFV: Mpulungu flavivirus
MSA: multiple sequence alignment
NGOV: Ngoye virus
PCA: principal components analysis
prM: pre-membrane
RT-qPCR: reverse transcriptase quantitative PCR
sfRNA: sub-genomic flaviviral RNA
SL: stem-loop
TBF: tick-borne orthoflaviviruse
TEM: transmission electron microscopy
UTR: untranslated region
XiFV: Xinyang flavivirus
xrRNA: exoribonuclease-resistant RNA
