# Supplementary material for: Xinyang flavivirus, from Haemaphysalis flava ticks in Henan Province, China, defines a basal, likely tick-only Orthoflavivirus clade
Source: J Gen Virol. 2024 May 29;105(5):001991. doi: 10.1099/jgv.0.001991 (PMC11165663; doi:10.1099/jgv.0.001991)
Supplement: Uncited Supplementary Material 1. [file jgv-105-01991-s001.pdf]

Supplementary Material: Wang and Cheng *et al.,* 2023 Xinyang flavivirus, from *Haemaphysalis flava* ticks in Henan province, China, defines a basal, likely tick-only *Orthoflavivirus* clade.

Supplementary table 1. Putative polyprotein cleavage sites of Xinyang flavivirus and closely related tick orthoflaviviruses. VSP, viral serine protease; HS, host signalase. XiFV (GenbankID: WGN99773.1), MPFV (GenbankID: BCL56285), NGOV (GenbankID: ABD64515.1), TBEV (GenbankID: ABS00284).

Cleavage site	Protease	Virus	Amino acid sequence
**VirC/CTHD**	**VSP**	XiFV:<br>MPFV:<br>TBEV:	81 TGRRR↓QRRGW 89<br>79 AGRRR↓ANTGQ 88<br>92 RGKRR↓SATDW 101
**AnchC/prM**	**HS**	XiFV:<br>MPFV:<br>TBEV:	104 GGTFA↓ARATM 113<br>97 SLALG↓AHVFM 106<br>112 GMTLA↓ATVRK 121
**Pr/M**	**Furin**	XiFV:<br>MPFV:<br>TBEV:	192 RIVERSLSVT 201<br>186 RIAERSLSVT 195<br>201 SRTRR↓SVLIP 210
**M/E**	**HS**	XiFV:<br>MPFV:<br>TBEV:	267 GPAYG↓TQCLG 276<br>261 APAYG↓TQCLG 270<br>276 APVYA↓SRCTH 285
**E/NS1**	**HS**	XiFV:<br>MPFV:<br>TBEV:	762 LGVGA↓DMACG 771<br>757 LGVGA↓DVACG 766<br>772 LGVGA↓DVGCA 781
**NS1/NS2A**	**?**	XiFV:<br>MPFV:<br>TBEV:	1110 GYVAA↓WVEAA 1119<br>1104 GFVYA↓WALEV 1114<br>1120 GLVRS↓MVVAD 1129
**NS2A/NS2B**	**VSP**	XiFV:<br>MPFV:<br>TBEV:	1343 RGSRR↓AAIGD 1352<br>1338 RAQRR↓AALGD 1347<br>1354 HRGRR↓SFSEP 1364
**NS2B/NS3**	**VSP**	XiFV:<br>MPFV:<br>TBEV:	1476 PRSQR↓ADLMG 1486<br>1473 PGNRR↓ADLVG 1482<br>1485 RTARR↓SDLVF 1494
**NS3/NS4A**	**VSP**	XiFV:<br>MPFV:<br>NGOV:<br>TBEV:	2099 MFFRK↓SASDV 2108<br>2091 QALRR↓SAGDI 2100<br>450 HALRR↓SAGDI 459<br>2106 ASGRR↓SFGDV 2115
**NS4A/2K**	**VSP**	XiFV:<br>MPFV:<br>NGOV:<br>TBEV:	2225 PGSQR↓GVLDN 2234<br>2217 PGGQR↓GVLDN 2226<br>576 PGGQR↓GVLDN 585<br>2232 AGKQR↓SSDDN 2241
**2K/NS4B**	**HS**	XiFV:<br>MPFV:<br>NGOV:<br>TBEV:	2248 GLVYC↓NEMGL 2257<br>2240 GLVYC↓NEMGL 2249<br>599 GMVYC↓NEMGL 608<br>2255 GLVAA↓NEMGF 2264
**NS4B/NS5**	**VSP**	XiFV:<br>MPFV:<br>NGOV:<br>TBEV:	2501 GDSRR↓GNAAV 2510<br>2949 RDGRR↓GAGHM 2503<br>853 RDGRR↓GAGHM 862<br>2507 SGSRR↓GGSDG 2516

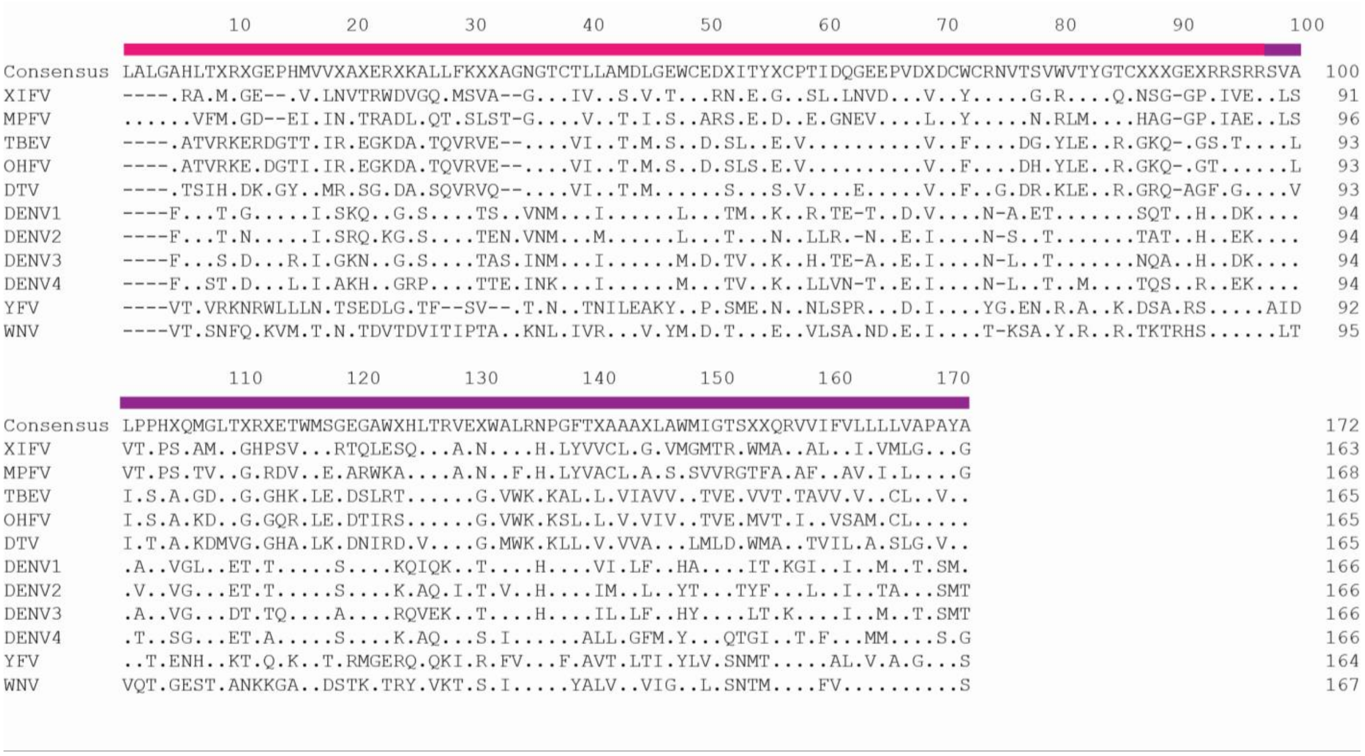

Supplementary figure 1. Alignment of the XiFV and MPFV prM protein with other *Orthoflavivirus* species. TBEV (GenbankID: ABS00284), DTV (GenbankID: AF311056_1), OHFV (GenbankID: QRI43507), DENV1 (GenbankID: AYP31257), DENV2 (GenbankID: QIJ58805), DENV3 (GenbankID: QIS48879), DENV4 (GenbankID: AVY51410), YFV (GenbankID: QGN18670), WNV (GenbankID: QKN22593). Dots indicate conserved residues. Domains of XiFV are shown by colour for premembrane as pink and membrane as purple.

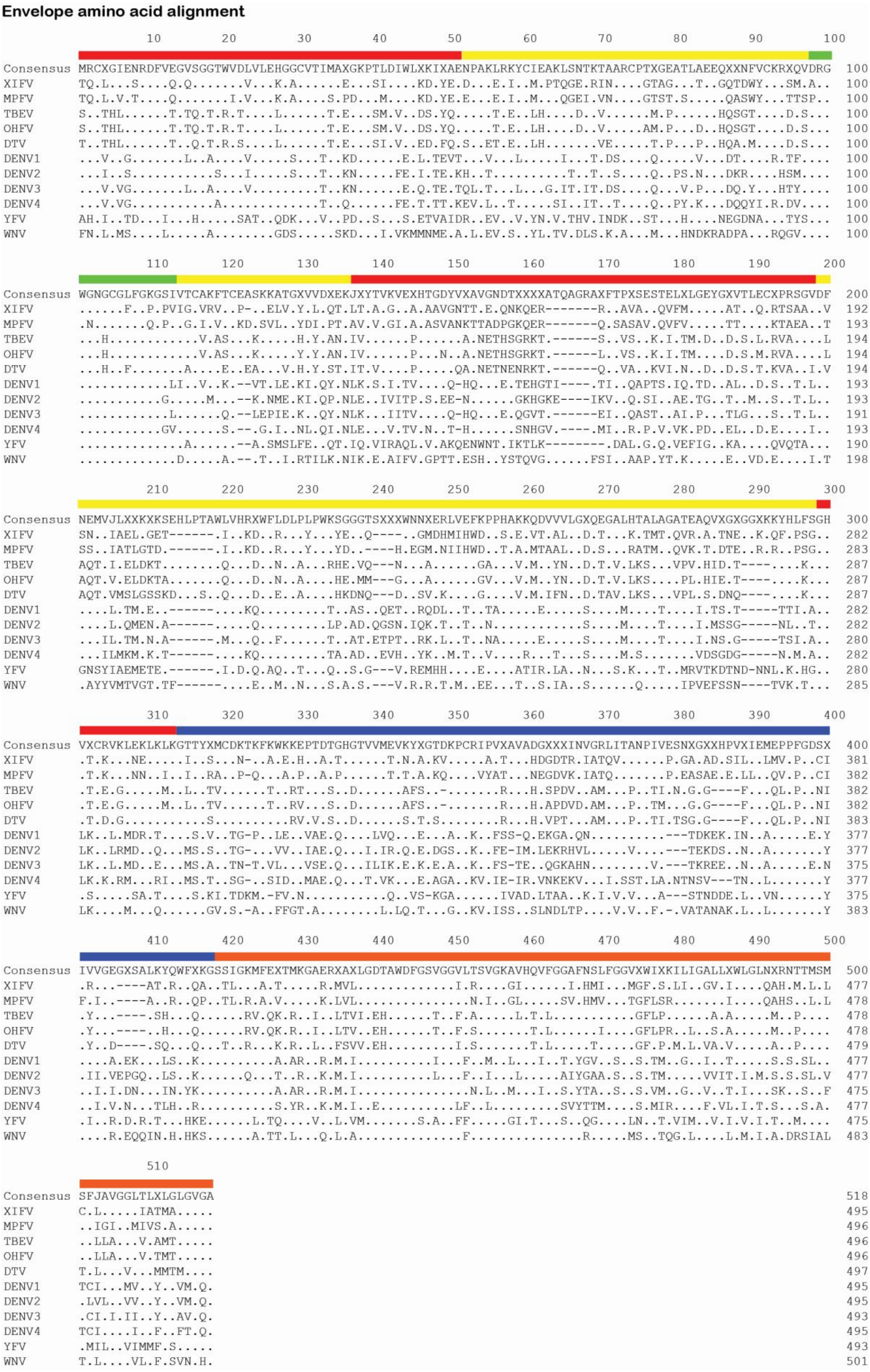

Supplementary figure 2. Alignment of the XiFV and MPFV E protein with other *Orthoflavivirus* species. TBEV (GenbankID: ABS00284), DTV (GenbankID: AF311056_1), OHFV (GenbankID: QRI43507), DENV1 (GenbankID: AYP31257), DENV2 (GenbankID: QIJ58805), DENV3 (GenbankID: QIS48879), DENV4 (GenbankID: AVY51410), YFV (GenbankID: QGN18670), WNV (GenbankID: QKN22593). Dots indicate conserved residues. Domains of XiFV are shown by colour for domain I – red, domain II – yellow, domain III – blue, and stem and transmembrane – orange. Fusion peptide is indicated in green.

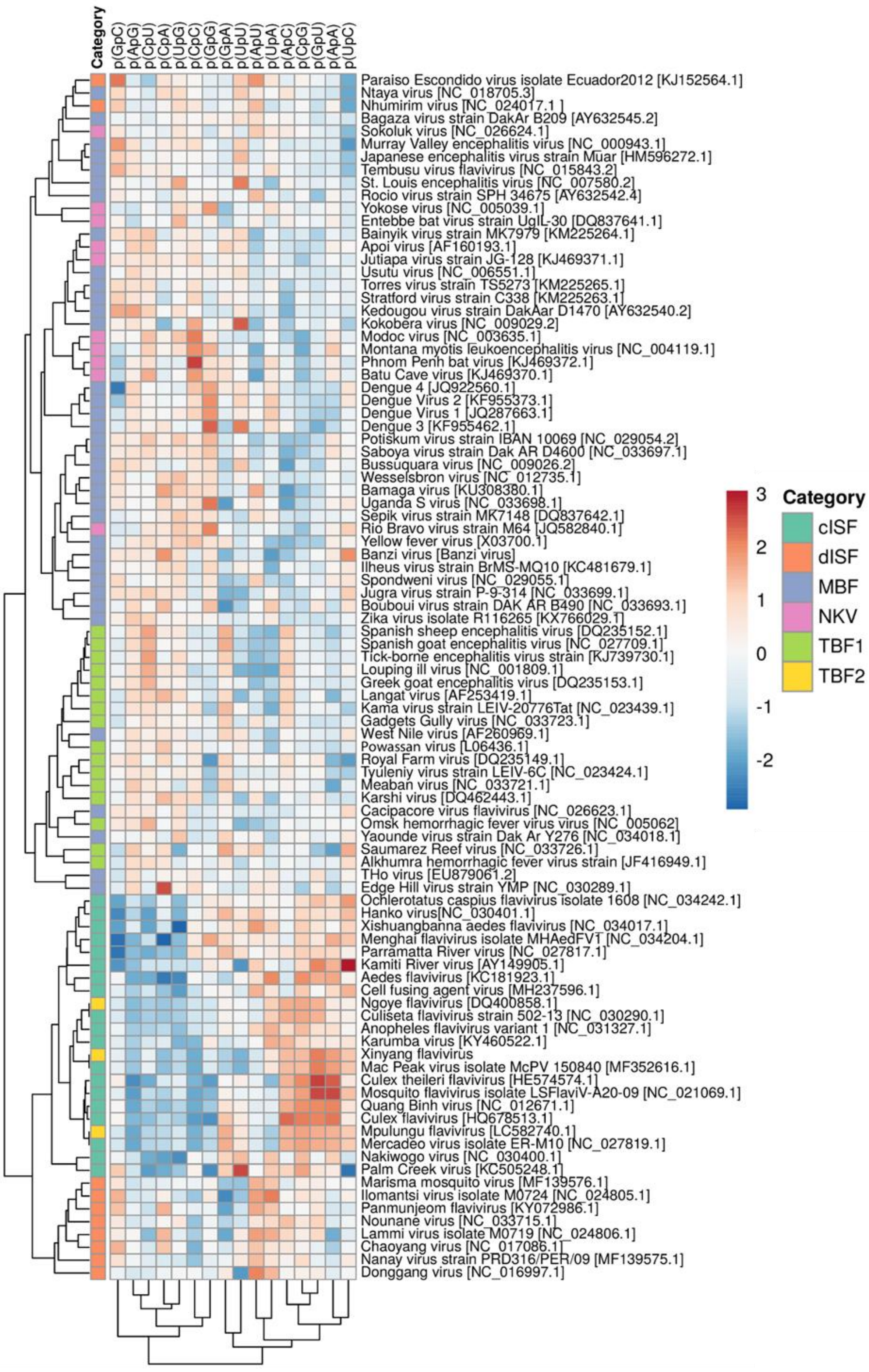

Supplementary figure 3. Hierarchical clustering analysis of dinucleotide composition of XiFV compared to other *Orthoflavivirus* species. Rows are centred, and unit variance scaling is applied to rows. Columns are clustered using Euclidean distance and complete linkage. Original values are $l_n(x + 1)$-transformed. Labels are as follows: TBF1: tick-borne orthoflavivirus group one, TBF2: tick-borne orthoflavivirus group two, MBF: mosquito-borne orthoflavivirus, dISF: dual-host associated or lineage two insect-specific orthoflavivirus, cISF: classical insect-specific orthoflavivirus.
